# Supplementary material for: Suppression of Ovarian Cancer Cell Proliferation Is Associated with Upregulation of Cell-Matrix Adhesion Programs and Integrin-β4-Induced Cell Protection from Cisplatin
Source: Cancers (Basel). 2025 Apr 27;17(9):1472. doi: 10.3390/cancers17091472 (PMC12070841; doi:10.3390/cancers17091472)
Supplement: Supplementary file 1 [file cancers-17-01472-s001.zip › cancers-3499327-supplementary.pdf]

Supplementary Materials

Suppression of Ovarian Cancer Cell Proliferation Is Associated with Upregulation of Cell-Matrix Adhesion Programs and Integrin-β4-Induced Cell Protection from Cisplatin

Sadaf Farsinejad, Daniel Centeno, Jan Savas-Carstens, Teagan Polotaye, Tonja Pavlovič, Pouria Babvey, Taru Muranen, Cezary Miedziarek, Piotr Jasiński, Elżbieta Dziabaszewska, Mikołaj Piotr Zaborowski, Pek Yee Lum, Laura A. Martin and Marcin P. Iwanicki

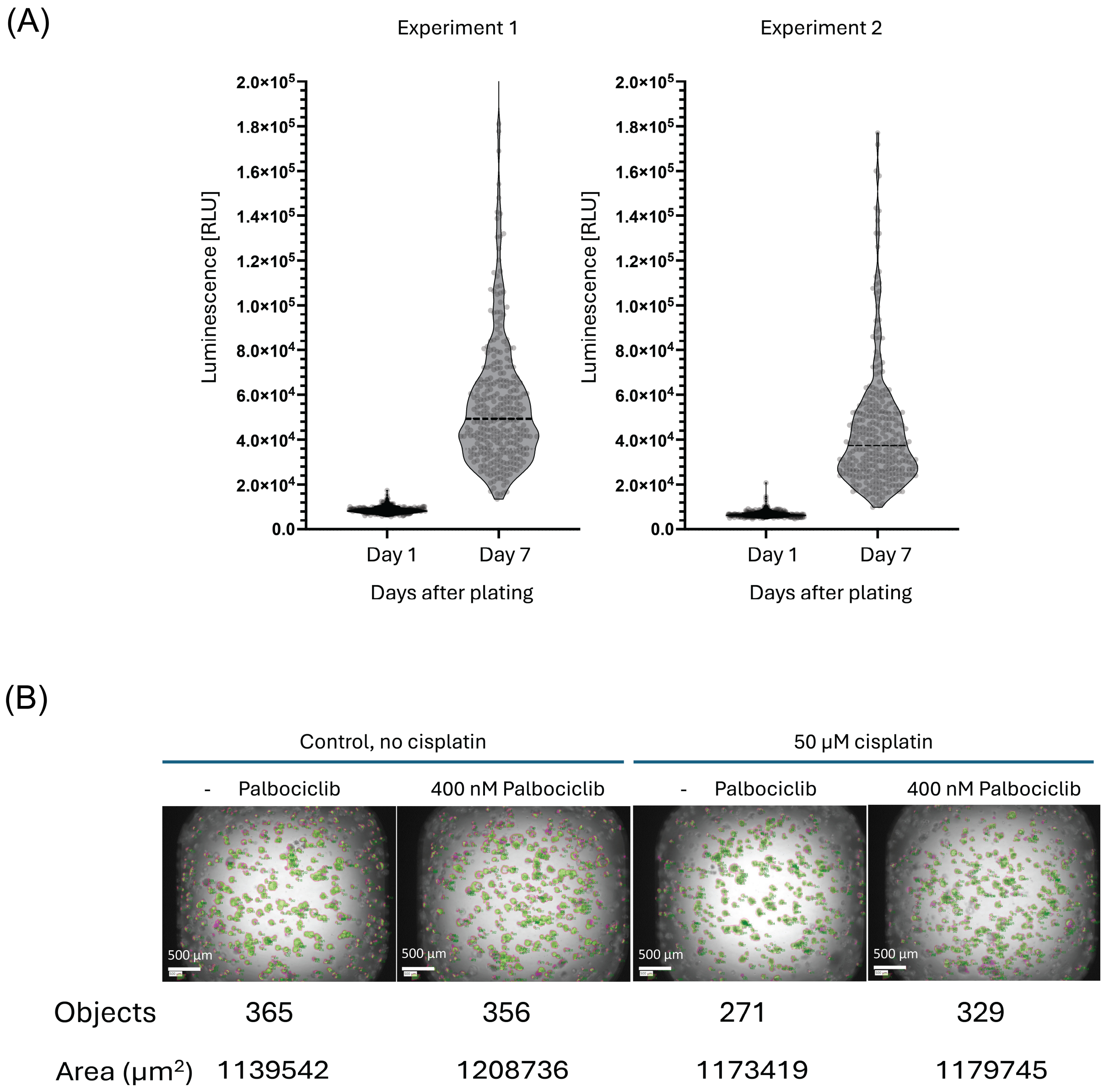

Figure S1. Organoid quantification in 384-well plates. (A) Violin plots from two independent experiments showing the distribution of luminescence values from CellTiter-Glow® 3D cell viability assays as a surrogate for cells/organoid quantification in each well of 384-well plates (308 plated wells total, represented by each dot). Single cell suspensions were plated in two separate plates (Day 1 and Day 7) at 1000 cells per well in 70% Cultrex BME solid domes for organoid formation and growth. CellTiter-Glow® 3D cell viability assays were performed 24h after plating (Day 1) to determine the variability at plating, and at Day 7 after plating to determine the number of organoids grown in each well at the time point at which cisplatin is added in organoid drug assays; (B) Image-

based organoid quantification in drug assays. Representative bright field images taken with a Keyence BZ-X810 microscope at day 12 after single cell plating and treated with the indicated drug(s) and doses or vehicle control. The Keyence Hybrid Cell Count Analysis Software was used for quantification of the organoids (objects) and total surface area occupied by organoids in the focal plane. Each identified object is masked (green) and quantified. The counts for individual objects as well as the total surface area occupied by all the counted objects is shown.

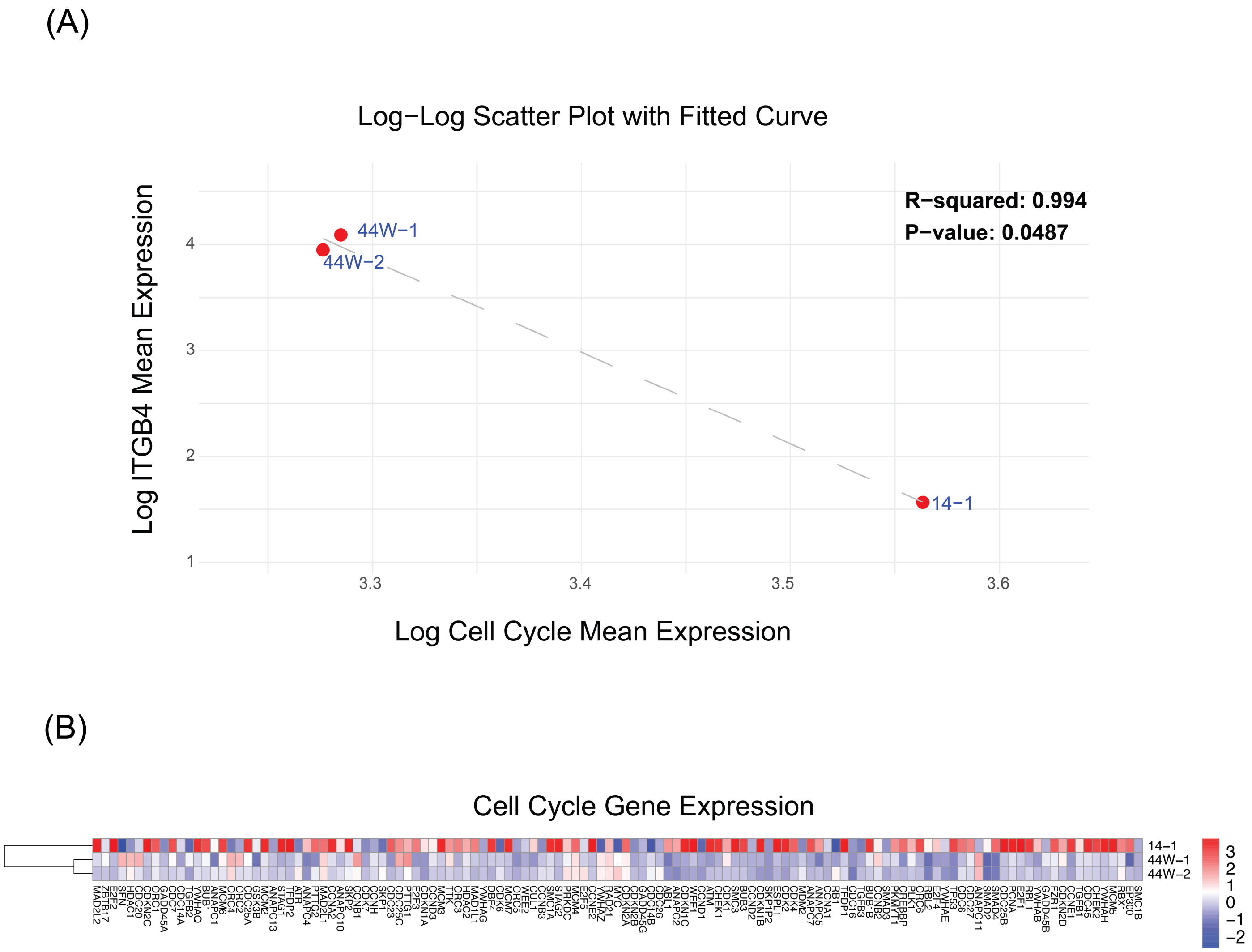

Figure S2. *ITGB4* mRNA expression versus cell cycle gene expression in PDOs. (A) Scatter plot of average ITGB4 mRNA expression relative to cell cycle gene expression in ovarian cancer PDOs (44W-1 and 44W-2), used in this study, and PDO14, which originates from a neuroendocrine tumor and was not included in our main analyses. The organoid with the lowest ITGB4 expression (PDO14) exhibits the highest levels of cell cycle genes; (B) Heatmap displaying the expression of cell cycle genes across the same three PDOs. PDO14 shows distinctly higher cell cycle gene expression relative to the ovarian cancer PDOs 44W-1 and 44W-2.

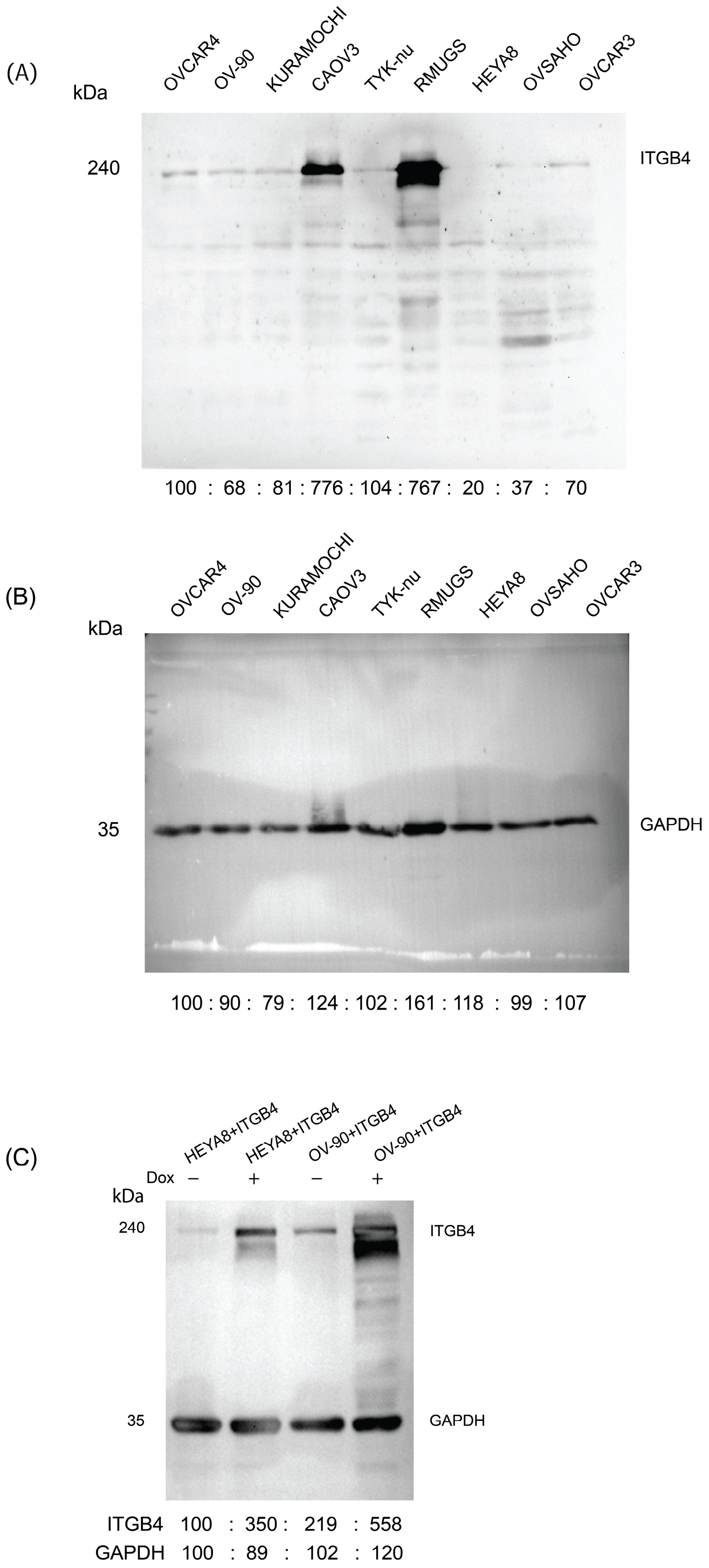

Figure S3. Uncropped Western blot images corresponding to the blots shown in Figures 3A and 7A. (A) Full blot for ITGB4 detection in ovarian cancer cell lines, corresponding to Figure 3A. Relative densitometry values (normalized to the first lane) are shown below; (B) Full blot for GAPDH loading control in the same cell lines, corresponding to Figure 3A. Relative densitometry values are shown below. (C) Full blot for ITGB4 and GAPDH expression in doxycycline-inducible HEYA8 and OV-90 cells, corresponding to Figure 7A. Relative densitometry values are shown below for each set of bands.
